# Mineral Content of Various Portuguese Breads: Characterization, Dietary Intake, and Discriminant Analysis

**DOI:** 10.3390/molecules24152787

**Published:** 2019-07-31

**Authors:** Álvaro Torrinha, Marta Oliveira, Susana Marinho, Paula Paíga, Cristina Delerue-Matos, Simone Morais

**Affiliations:** 1 REQUIMTE-LAQV, Laboratório de Química Aplicada, Faculdade de Farmácia, Universidade do Porto, Rua de Jorge Viterbo Ferreira, 228, 4050-313 Porto, Portugal; 2 REQUIMTE-LAQV, Instituto Superior de Engenharia do Porto, Instituto Politécnico do Porto, R. Dr. António Bernardino de Almeida 431, 4249-015 Porto, Portugal

**Keywords:** breads, mineral composition, high-resolution continuum source atomic absorption spectrometer, daily intake, heath risks and benefits, chemometric discrimination

## Abstract

The chemical composition and daily mineral intake (DMI) of six macro (calcium, magnesium, sodium, potassium, phosphorous, and chloride) and four microminerals (copper, iron, manganese, and zinc) were determined in four types of Portuguese breads (white wheat, maize, wheat/maize, and maize/rye breads). Samples were processed with microwave assisted digestion and mineral composition was determined with a high-resolution continuum-source atomic absorption spectrometer with flame and graphite furnace. Bread contributes to an equilibrated diet since it is rich in several minerals (0.21 mg/100 g of copper in wheat bread to 537 mg/100 g of sodium in maize/rye bread). Maize/rye bread presented the highest content of all minerals (except phosphorous and chloride), while the lowest levels were mainly found in wheat bread. Median sodium concentrations (422–537 mg/100 g) represented more than 28% of the recommended daily allowance, being in close range of the maximum Portuguese limit (550 mg/100 g). Maize/rye bread exhibited the highest DMI of manganese (181%), sodium (36%), magnesium (32%), copper (32%), zinc (24%), iron (22%), potassium (20%), and calcium (3.0%). A Principal Component Analysis (PCA) model based on the mineral content allowed the differentiation among white wheat, maize, and maize/rye bread. Zinc, magnesium, manganese, iron, phosphorus, potassium, copper, and calcium proved to be good chemical markers to differentiate bread compositions.

## 1. Introduction

Bread is considered a staple food for being a major part of the human diet. Nutritionally balanced, bread is part of an equilibrated and healthy diet and is a good source of energy, rich in carbohydrates, namely starch, and with little fat content. Also, it is a source of dietary fiber and contains vitamins B and E, and proteins [1,2]. In Portugal, bread is consumed on a regular basis, therefore bread is among the top five products produced (410,992,024 ton in 2016) by Portuguese food industries being the main product sold by the bakery industry, surpassing the sales of pastry shops [3].

Wheat cereal is the main food crop produced worldwide and the majority is used for leavened bread production [4]. Portugal is no exception, where the wheat bread produced was more than 50% of total bread [5]. Cereal grain structure is constituted by three features: endosperm, germ, and bran with different nutrient composition. For refined bread production, only the endosperm is used making white bread nutritionally poorer than wholemeal breads. Although there is a recognition that wholemeal breads have nutritional health benefits over white bread (or refined flour bread), the latter is still preferred by the Portuguese population [6] and worldwide consumers [7]. In European countries, the consumption of white bread is about 117 g/person/day, being only beaten by Middle Eastern countries (215 g/person/day) [7].

Regarding minerals, bread also constitutes an important source of macro and micro elements [1,2,6,8,9,10]. In the human body, minerals play structural, catalytic, and regulatory functions. They activate enzymes and regulate pH fluids for metabolic reactions and cellular osmotic exchanges [11]. Even nowadays, mineral deficiencies are still common in developing countries. Iron deficiency is probably the most common nutritional deficiency in the world, but zinc deficiency is also very frequent. Mineral bioavailability of cereal-based diets of developing countries is probably the main cause for these deficiencies [12]. Multicereal and/or pseudocereal mixtures have been increasingly introduced in the bakery industry, principally in the production of bread to increase its nutritional value [6,9,13,14,15,16,17]. Many authors reported that white wheat partial replacement and/or enrichment with other cereals and/or pseudocereals strongly increased the nutritional value of bread, in particular, the proteins, minerals, healthy fats and antioxidants, and fiber content [13,14,15,16,17,18,19]. Recently, some works have emerged regarding the supplementation of wheat flour with other vegetable products, leafy vegetable powders [8], and fish filleting residues [18]. Mineral content (mainly concerning calcium (Ca), magnesium (Mg), sodium (Na), potassium (K), phosphorous (P), copper (Cu), iron (Fe), manganese (Mn), and zinc (Zn)) in white wheat bread from diverse geographical origins (Africa: Nigeria; Asia: Saudi Arabia, Egypt, India; Europe: Bulgaria, Finland, Serbia, Spain, Poland, Portugal, Turkey; America: Brazil, Chile, Ethiopia, Mexico, United States of America) is well reported in literature [1,6,8,13,14,15,16,17,18,19,20,21,22,23,24,25,26,27,28,29] (Table 1). Also, studies regarding the mineral content of maize [9,12,21,25] and rye [6,10,20,21,26,27] breads were found in the literature but to a much lesser extent; no data was found for bread prepared with mixtures of maize and rye flours (Table 1). Frequently, bread has a high content of Na, since sodium chloride is added to improve flavor and texture but with possible negative side effects on human health when consumed in excess. Portugal is one of the countries that adds high quantities of this salt in bread and to impose its reduction, authorities lowered the limit of salt added to 1.4 g per 100 g of bread, i.e., 550 mg/100 g of Na [30]. So far, scarce information exists regarding the mineral content of widely consumed Portuguese breads [6,21,26,31]. Furthermore, regardless of the country, studies that simultaneously include the characterization of several macro- and microelements in bread are still limited [9,10,13,16,17,20,21,23,28]. Regarding Portugal, there are only three studies that assessed the mineral content of breads [6,21,26], with only one including several macro- and microminerals [21].

Thus, the main aim of the present work was to determine the macromineral (Ca, Mg, Na, K, P, and chloride (Cl)) and micromineral (Cu, Fe, Mn, and Zn) composition of different types of breads typically consumed in Portugal: white wheat bread, maize bread, a bread prepared with a mixture of wheat and maize flours, and a bread of maize and rye flours (called “Broa de Avintes” and traditionally made in the Oporto region of Avintes). Other goals were (i) to evaluate if the levels of Na in bread comply with the maximum amount imposed by Portuguese legislation, (ii) to assess the daily mineral intake (DMI) of each element for the consumption of the breads based on the recommended daily allowances (RDA) and adequate intake (AI), and (iii) to explore, for the first time, the mineral content to determine chemical descriptors and interrelationship patterns in breads.

## 2. Results and Discussion

### 2.1. Mineral Content

**Figure 1:**
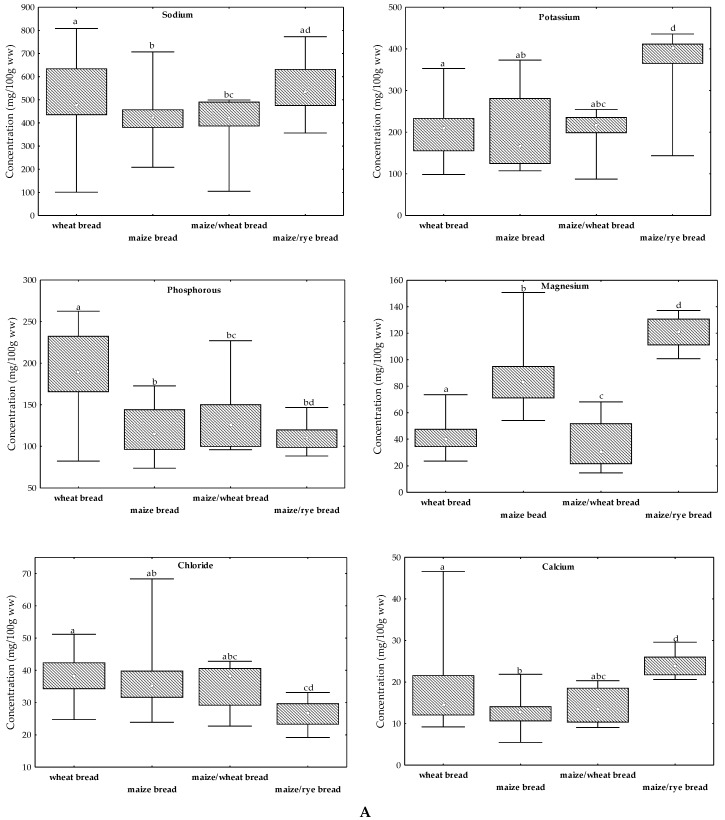

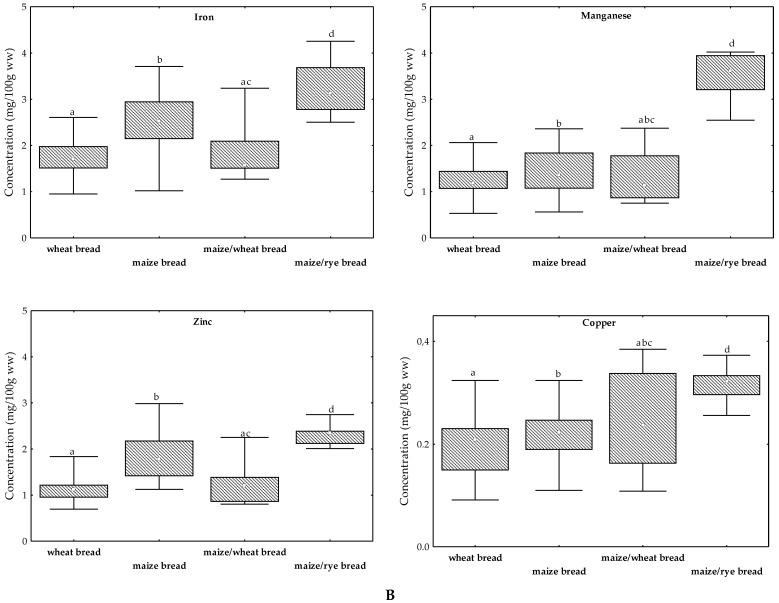
Concentrations (□ Median, 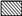 25–75% and 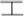 range, mg/100 g ww) of (**A**) macroelements and (**B**) microelements in breads. Each letter (a−d) corresponds to a type of bread (a, wheat bread; b, maize bread; c, maize/wheat bread; d, maize/rye bread). The same letter in a box plot indicates that the given medians are not statistically different (*p* > 0.05).

The mineral composition of the studied breads is displayed as median, 25 and 75 percentiles, minimum and maximum amounts in Figure 1 and organized based on elements’ essentiality as macroelements (Figure 1A) and microelements (Figure 1B). The attained macroelement profiles (Figure 1A) for wheat bread, maize bread, and maize/wheat bread were the same and were as follows: Na > K > P > Mg > Cl > Ca. For maize/rye bread, Mg was higher than P content presenting the following order: Na > K > Mg > P > Cl > Ca (Figure 1A). Na was the most predominant element in all breads compared with the other elements, with median values ranging from 422 mg/100 g ww (maize bread) to 537 mg/100 g ww (maize/rye bread). This was expected since Na is added during the breadmaking process as table salt, also explaining the high variability of results reported by other authors [6,13,21,26,27,28] (Table 1). Refined sea salt is the most commonly used salt in Portuguese bakeries, containing more than 99% sodium chloride; some additives such as whitening and anti-caking agents (such as ferrocyanide salts, sodium silicoaluminate, and magnesium carbonate) may also be added to maintain humidity levels [31]. Table salt, sodium chloride, is by weight approximately 40% Na and 60% Cl. However, this proportion in not observed in breads because of other sources of Na, such as baking soda (sodium bicarbonate) which is added to bread doughs. On the other hand, Ca exhibited the lowest amount of the analyzed macroelements in all breads with median values ranging from 12.7 mg/100 g ww (maize bread) to 23.8 mg/100 g ww (maize/rye bread) confirming the fact that breads have a small content of Ca (Figure 1A). The median concentration of the other macroelements varied from 166 mg/100 g ww (maize bread) to 403 mg/100 g ww (maize/rye bread) for K; 111 mg/100 g ww (maize/rye bread) to 189 mg/100 g ww (wheat bread) for P; 30.5 mg/100 g ww (maize/wheat bread) to 121 mg/100 g ww (maize/rye bread) for Mg; and 26.6 mg/100 g ww (maize/rye bread) to 38.4 mg/100 g (maize/wheat bread) for Cl (Figure 1A).

The content of the elements that are required in small amounts by the human body, but nevertheless essential and designed as microelements, are described by the following order: Fe > Mn ≈ Zn > Cu for wheat bread; Fe > Zn ≈ Mn > Cu for maize bread; Fe ≈ Mn ≈ Zn > Cu for maize/wheat bread; Mn ≈ Fe > Zn > Cu for maize/rye bread (Figure 1B). Fe was the microelement existing in highest amount in wheat, maize, and maize/wheat breads with median values ranging from 1.57 mg/100 g ww (maize/wheat bread) to 2.52 mg/100 g ww (maize bread). Mn was the microelement present in highest levels in maize/rye bread (3.62 mg/100 g ww); in the other types of bread, Mn concentrations varied between 1.13 mg/100 g ww (maize/wheat bread) to 1.37 mg/100 g ww (maize bread); Figure 1B. Cu was the least abundant mineral in all breads with values varying between 0.21 mg/100 ww (wheat bread) and 0.32 mg/100 g ww (maize/rye bread). Zn content was comparable to that of Mn in wheat, maize, and maize/wheat breads (1.13–1.77 mg/100 g), while being also similar to Fe in maize/wheat and maize/rye breads (Figure 1B). In general, the bread made with maize and rye flours had the highest content of macroelements (except for P and Cl) and microminerals when compared to the other breads (Figure 1). These findings are explained by the fact that rye grains have higher mineral content than white wheat and maize grains [20,27]. Therefore, white wheat bread enriched with mixtures of other cereals (such as maize and/or rye) and pseudocereals exhibit increased mineral levels, as previously reported by other authors [13,14,15,16,17]. Maize/rye bread was revealed to be statistically different from the other breads for all elements (*p* < 0.05) except for Na, P, and Cl, wherein similarities occurred with wheat (*p* = 0.19), maize (*p* = 0.31), and maize/wheat bread (*p* = 0.05), respectively (Figure 1). Mg concentrations can be used to differentiate (*p* < 0.05) all breads. Also, Fe and Zn allowed the distinction (*p* > 0.05) of breads, except for wheat and maize/wheat breads. Since these two bread types had wheat in their composition, they exhibited similarities (*p* > 0.05) concerning Ca, K, Cl, Cu, Fe, Mn, and Zn amounts (Figure 1). Furthermore, the same behavior was observed for maize and maize/wheat breads for most of the analyzed elements (Ca, Na, K, P, Cl, Cu, and Mn). In line with these findings, wheat bread was significantly different (*p* < 0.05) from maize bread for all the minerals except for K and Cl (Figure 1).

Data concerning mineral content in breads (2000–2017) are reviewed in Table 1. The reported information is mostly related to wheat bread and also, in most of the reports, a very limited number of samples and/or types of bread were characterized. No literature studies were found regarding minerals in maize/rye bread mixtures.

Despite the median concentrations of Mg, Na, K, and P in Portuguese white wheat bread being slightly higher than the levels reported in the literature for the same type of bread (except for the works performed by Castanheira et al. [6], Martins [21], and Watnick [27] for Na, Tuncel et al. [19] for K, and Bilgiçli and İbanoğlu [14] for P (Table 1), the range of concentrations were globally within the reported values except for some Spanish, Polish, Serbian, and Egyptian white wheat bread [13,16,17,23]. Median levels of Ca in Portuguese wheat bread (14.5 mg/100 g ww) were predominantly lower than the concentrations reported by other authors, except for the concentrations presented in Brazilian (2.5 mg/100 g ww; [18]) and Indian (11.6 mg/100 g ww; [24]) wheat breads (Table 1). Regarding the levels of microelements, the achieved median concentrations of Cu in Portuguese wheat bread were lower by 9 to 29% than the levels reported in Turkish [14] and Egyptian [17] wheat breads but were predominantly higher (33–95%) than in Chilean, Finnish, Serbian, Polish, and Spanish breads [13,16,20,22,23]. Limited information is available regarding Mn mineral content in bread (Table 1). Median levels of Mn in wheat bread (1.20 mg/100 g ww; Figure 1B) were two (Finland; [20]) to 40 (Spain; [13]) times higher than the concentrations reported in the literature (Table 1); there is no information for comparison regarding the mineral content of Mn in Portuguese bread (Table 1). Fe and Zn median levels were in general lower than the values presented for Brazil [18], USA [27], and Ethiopia [25]. The findings of this study for the majority of the elements determined in wheat bread samples were in close range of the levels reported in Food Composition Tables of Portuguese [21] and United States of America [27] wheat bread products. Reported concentrations of Ca in wheat bread were 5.5 [27] and 2.3 times higher [21] than in the analyzed wheat bread samples (14.5 mg/100 g ww; Figure 1A). The achieved levels of Fe in wheat bread (1.71 mg/100 g ww; Figure 1B) were in close range of the concentrations presented in the Portuguese Food Composition Table [21], but about two times lower than the levels reported in the American Food Composition Table [27].

Only two studies were found in the literature regarding mineral content in maize/wheat bread [6,9]. Castanheira et al. [6] determined Na and K content in wheat, rye, and maize/wheat breads marketed in Portugal. Maize/wheat bread presented mean levels of 629 mg/100 g ww of Na and 253 mg/100 g ww of K which were slightly higher than the median concentrations found in this study (456 mg/100 g ww for Na and 217 mg/100 g ww for K; Figure 1A). Rybicka and Gliszczyńska-Świgło [9] evaluated the mineral content in different mixtures of Polish maize bread with wheat starch, linseed, sunflower, and pumpkin seeds. The addition of different seeds to the mixture of maize and wheat starch increased the mineral levels of Mg, K, Cu, Fe, Mn, and Zn (Table 1). Limited data exist on the mineral content of rye bread [6,10,20,21,26,27]; none exists regarding the mineral content of maize/rye bread mixtures, thus results obtained for maize/rye bread can only be compared with rye and/or maize breads. Szymczycha-Madeja [10] reported mineral levels of Mg, Cu, Fe, Mn, and Zn in Polish rye bread that was similar to the concentrations found in this study for maize/rye bread. 

The variability of results found in the literature is somehow explained by extrinsic and intrinsic factors. The amount of minerals found in wheat bread is somehow dependent on the percentage of extraction in white bread production (or refined flour). The designation of white bread occurs when the extraction reaches 75% or lower. Decreasing the extraction percentage leads to a decrease of the mineral levels in bread [32]. Generally, the concentrations of Ca reported in most of the literature found were much higher than the levels of Ca presented herein since, in some countries, the addition of nutrients, including Ca and Fe, is required by law to restore the content lost in the milling process [2]. Another reason for fortification is the fact that bread prepared with cereal grains, principally wheat, presents low mineral content [6,20,26,27]. Also, soil composition, use of fertilizers, and geographical location of the cultivars may influence the mineral content in grains [20].

### 2.2. Dietary Intake and Health Assessment

Minerals play an important role in human health with structural, regulatory and catalytic functions in the body and are essentially required in the human diet [11]. To assess the dietary importance of bread relative to mineral intake, DMI percentages were estimated based on the consumption of 100 g of bread and on the recommended daily allowances (RDA) and adequate intakes [33,34]. Results are presented in Table 2. The DMI results obtained for the four types of bread, obey to the following order: Mn > Na > P > Cu > Fe > Zn > Mg ≈ K > Cl > Ca for wheat bread; Mn > Na > Mg ≈ Cu > Fe ≈ Zn > P > K > Cl > Ca for maize bread; Mn > Na > Cu > P > Zn > Fe > K > Mg > Cl > Ca for maize/wheat bread; and Mn > Na > Mg = Cu > Zn > Fe > K > P > Cl ≈ Ca for maize/rye bread. Mn promoted the highest DMI by consumption of all breads with median values ranging from 60% (wheat bread) to 181% (maize/rye bread). Wheat, maize, and maize/wheat bread samples presented maximum DMI that slightly exceeded the RDA value of 2 mg/day [33,34]; Mn content in maize/rye bread samples exceeded the RDA value by up to two times (127 to 201%; Table 2). Therefore, bread may constitute an important source of Mn in the human diet. Daily consumption of 100 g of bread represented an ingestion of 28% (maize bread) to 36% (maize/rye bread) of the Na DMI (Table 2). Belz, Ryan, and Arendt [35] also reported that bread contributes up to 26% of Na daily intake in Irish adults. Median Na levels varied between 422 mg/100 g ww in maize bread to 537 mg/100 g ww in maize/rye bread (Figure 1A), values that are slightly lower than the maximum concentration allowed by Portuguese legislation (550 mg/100 g of Na; [30]). A total of 28, 25, and 11% of white wheat, maize/rye, and maize bread samples, respectively, presented Na concentrations exceeding that limit. All the samples of maize/wheat bread presented Na levels (median of 456 mg/100 g; range: 379–499 mg/100 g) that were below the guideline (Figure 1A). Na is principally added in the form of sodium chloride, but also as sodium bicarbonate, to bread and other daily products (principally the processed ones) to improve their flavor, shelf-life, and texture. Several works already evaluated the reduction and/or replacement of sodium chloride by other compounds, such as potassium chloride, and, despite the fact that no significant disadvantages were reported, during the bread production process, several adverse effects were registered in the flavor, shelf-life, and in the rheological characteristics of bread [35]. High ingestions of salt are directly associated with elevated blood pressure values throughout life and strongly contribute to increase the risk of cardiovascular diseases [36]. The amount of salt added to bread changes from bakery to bakery (up to 1400 mg/100 g; [30]) and, thus, it is expected to vary widely within and between countries. Therefore, more studies regarding the Na content in different types of bread at a national and international level are needed. In 2013, the World Health Organization member states agreed to reduce the populations’ Na intake by 30% by 2025 [37]. Despite bread being one of the major sources of salt (up to 20–30% of the diet in some countries), the reduction of salt in bread alone will not have the necessary impact in the diet of the populations to achieve the WHO 2025 target [38]. Thus, mechanisms of monitoring at a national level and programs of industry engagement are needed to help the food industry to reduce salt levels.

Ingestion of bread contributed to 3.9–40%, 11–38%, and 4.4–22% of Mg, P, and K DMI, respectively (Table 2). Regarding the DMI of microelements, bread contributed to 9.1–38% of Cu, 6.8–30% of Fe, 7.0–30% of Zn, and 2.4–6.4% of Cl (Table 2). Data show that breads contribute poorly to daily Ca intake (0.7–5.8%). A diet rich in cereal-based products with low Ca content may contribute to long-term development of bone related problems. It is known that Ca bioavailability decreases through urinary excretion when high Na intake occurs [2]. Therefore, an equilibrated and healthy diet should include important sources of Ca (e.g., milk derivatives).

According to the European commission [33], a nutrient amount from a 100 g portion of a given food that reaches 15% of the RDA value is considered a significant quantity. Therefore, maize/rye bread is a valuable source of macro (Mg, Na, and K) and micro elements (Cu, Fe, Mn, and Zn) compared with the other types of bread considered in this study (Table 2). Maize bread constitutes an important source of Mn, Na, Mg, Cu, Zn, Fe, and P, thus contributing with higher intakes of Mn, Mg, Zn, and Fe compared with the mixture of maize/wheat bread. White wheat bread presented the lowest intakes of Cu and Zn comparatively with other breads (Table 2). Overall, bread is a good source of essential minerals; however, the amount absorbed by the organism cannot be predicted due to the presence of inhibitors in cereal based products such as phytates. Phytic acid reduces the bioavailability of Fe, Zn and Ca since it has high chelating activity [32]. Recently, Iglesias-Puig et al. [15] proved that whole quinoa flour is a good substitute for wheat flour in the preparation of bread by increasing its nutritional value and also found that the use of bifidobacterial phytases contributed to increase the DMI and the bioavailability of the minerals present in bread. Further complementary studies are required to perform a more comprehensive characterization.

### 2.3. Principal Component Analysis

**Figure 2:**
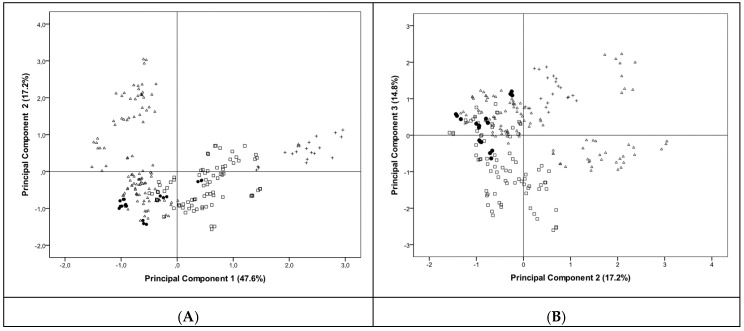
Principal component (PC) analysis applied to the four types of bread studied (Δ—wheat; □—maize; ●—maize/wheat; +—maize/rye) based on Mg, Ca, K, P, Cu, Fe, Mn, and Zn: (**A**) PC1 vs. PC2 and (**B**) PC2 vs. PC3.

The pattern recognition technique, Principal Component Analysis (PCA), was applied to the four types of characterized bread in order to identify chemical descriptors. The achieved PCA model allowed the extraction of three principal components with eigenvalues ≥1.2 (PC1, PC2, PC3) and fulfilling the Kaiser–Meyer–Olkin (KMO) sampling adequacy test (KMO = 0.75). Altogether the three PCs represented 80% of the original data. Data presented in Figure 2A is explained by 65% of total variance from the first two components (PC1 versus PC2), based on the following eight elements: Mg, Ca, K, P, Cu, Fe, Mn, and Zn (Na and Cl presented KMO < 0.5 and therefore were not considered in the model). Globally, the separation of maize/rye bread and to a lesser extent, wheat bread from the other types can be observed. The first component comprising 47.6% of variance clearly separated the maize/rye bread from the other breads and was strongly influenced by the contents of Zn (PC1 loading = 0.86), Mg (PC1 loading = 0.84), Mn (PC1 loading = 0.83), Fe (PC1 loading = 0.82), and Cu (PC1 loading = 0.67) in that bread. PC2 represented 17.2% of the total variance and was able to separate wheat bread from the other breads, being mostly influenced by the content of Ca (PC2 loading = 0.81) and P (PC2 loading = 0.65). These findings are in line with the significantly higher median levels of Zn, Mg, Mn, Fe, and Cu presented by maize/rye bread comparatively with the other three types of breads (*p* ≤ 0.05).

Regarding white wheat bread, a significantly higher content of P than in the other breads was found (189 mg/100 g versus 115 mg/100 g for maize bread, 126 mg/100 g for maize/wheat bread, and 111 mg/100 g for maize/rye bread; Figure 1A; *p* < 0.05). Ca but also K, Fe, Mn, Cu, and Zn levels in wheat bread were significantly different from the other breads, except from maize/wheat samples (Figure 1; *p* < 0.05). Data from PC2 and PC3 represented 32% of the total variability (Figure 2B). PC3 represented 14.8% of total variance and was strongly influenced by the mineral content of K (loading = 0.70). This factor allowed the discrimination between maize/rye from maize bread samples while PC2 allowed the differentiation between wheat bread from the other type of breads (Figure 2B). The median concentrations of K in wheat and maize/rye breads were significantly different between each other (209 mg/100 g versus 403 mg/100 g; *p* < 0.05), although the K levels in wheat bread were similar to maize and maize/wheat bread samples (209 mg/100 g versus 166 mg/100 g and 217 mg/100 g; *p* > 0.05; Figure 1A). Figure 2B permitted a better separation of maize bread samples than Figure 2A. The attained PCA model allowed the discrimination between three groups of bread (wheat, maize, and maize/rye bread) based on their mineral content (Figure 2). Levels of Zn, Mg, Mn, Fe, P, K, Cu, and Ca can be used as chemical markers to identify different types of flours that constitute breads. Previously, Ackura and Kokten [39] evaluated the variability of some mineral contents in wheat genotypes during two successive growing seasons and found that Zn, Mn, and Fe were the most representative of the overall mineral content and presented the highest discriminating power. Thus, some specific macro and/or microminerals can be particularly important to serve as chemical descriptors of traditional/regional breads and to identify breads with a higher nutritional value.

## 3. Materials and Methods

### 3.1. Sampling

Several sampling campaigns were randomly done in different bakery shops, markets, supermarkets, and hypermarkets from the Oporto metropolitan region (north of Portugal; second largest metropolitan area in the country). Labelled data regarding bread composition was registered. A total of 82 fresh bread samples were collected and characterized regarding the type of flour and/or mixtures of flours used in its preparation. The collected samples were divided into four different groups: wheat bread, maize bread, maize/wheat bread, and maize/rye bread. All the samples were cut into small pieces and mechanically ground with a Bosch blender (750 w, MSM7400, Slovenia) until a homogeneous mixture was obtained. A small portion of bread sample (around 100 g) was stored in plastic bags and frozen (−20 °C) until analysis. An MLS moisture analyzer (Kern; Balingen, Germany) was used for the determination of moisture in all bread samples.

### 3.2. Reagents

Mineral stock solutions (1000 mg/L) of Ca, Mg, Cl, Cu, Fe, Mn, and Zn, were obtained from Panreac (Barcelona, Spain) while K, Na, and P standards were obtained by potassium chloride (99.5%, Riedel-de Haën, Seelze, Germany), sodium chloride (99.8%, Riedel-de Haën, Seelze, Germany), and potassium dihydrogen phosphate (99.5%, Riedel-de Haën, Seelze, Germany) dissolution in ultrapure water (resistivity of 18.2 MΩcm^−1^, Millipore, Molsheim, France), respectively. Standard solutions and samples were prepared and diluted with ultrapure water acidified with 1% (*v*/*v*) suprapur nitric acid (65%; Sigma–Aldrich, Steinheim, Germany). A solution of cesium chloride (≥99.5%; Sigma–Aldrich, Steinheim, Germany) was used as flame ionization chemical suppressor. The matrix modifiers of 1% *v*/*v* Mg(NO_3_)_2_ and 0.1% *v*/*v* Pd(NO_3_)_2_/0.05% *v*/*v* Mg(NO_3_)_2_ were prepared daily and used for mineral analysis of Fe and Mn in the graphite furnace, respectively. A color development reagent was prepared with the addition of ammonium molybdate tetrahydrate (99.0%, Merck, Darmstadt, Germany) and ammonium metavanadate (99.0%, Merck, Darmstadt, Germany). This solution was used in the determination of P according to 4500-P standard procedure [40]. A solution of silver nitrate and sodium chloride purchased from Panreac (Castellar del Vallès, Barcelona, Spain) was used in the determination of chloride according to the Portuguese standard procedure NP-831/1970. All glass and polyethylene materials were washed and immersed overnight in a 10% (*v*/*v*) nitric acid solution, rinsed with ultrapure water and dried before use.

### 3.3. Mineral Composition

Homogenized milled samples were accurately weighted (~1.3 g) to Teflon vessels with an analytical balance (MS105, Mettler Toledo, Switzerland) and dried in a microwave Mars-X 1500 W (Microwave Accelerated Reaction System, CEM Mathews, NC, USA; equipped with a pressure and temperature sensors) until three reproducible weight values were obtained. An aliquot of 10.0 mL of suprapur nitric acid (65%) was added to ca. 0.2 g of dry sample (accurately weighed) in each vessel and a microwave assisted digestion (MAD) was performed for 35 min at 185 °C according to the procedure previously validated by Vieira et al. [41]. After digestion and cooling to room temperature, samples were transferred to polypropylene tubes and frozen at −20 °C until analysis. For each bread sample, three independent assays were performed.

The mineral composition of bread samples was determined with a High-Resolution Continuum Source Atomic Absorption Spectrometer (HR-CS-AAS, Analytik Jena ContrAA 700, Berlin, Germany) equipped with a xenon short-arc lamp XBO 301 (GLE, Berlin, Germany) operating in a hot-spot mode as a continuum radiation source, flame (AS 52 S) and graphite furnace (MPE60) autosamplers, and with a nominal power of 300 W operating in a hot-spot mode as a continuum radiation source. HR-CS-AAS has an advanced simultaneous background correction that allows the automatic elimination of lamp flicker noise and continuous background absorption. Visualization of the spectral environment of the selected analytical line at a high resolution allows avoiding spectral interferences.

Levels of Ca, Mg, Na and K were quantified on a flame mode with an air-acetylene (Air Liquid, Portugal) oxidizing flame for atomization. Concentrations of Cu, Fe, Mn, and Zn were determined with the Graphite Furnace module equipped with an MPE60 autosampler (Analytic Jena, Germany) on transversal and pyrolitically coated graphite tubes with integrated platform and with the use of argon as inert gas. The optimum characteristics used in the mineral analysis of bread samples, namely wavelength, acetylene/air flow, and height of burner for flame atomization and the graphite furnace optimized parameters, were adapted from previous works of this research group [41,42,43] and are presented in Table 3. P content was determined in a dual beam UV visible spectrophotometer (Evolution 300, Thermo Scientific, Waltham, MA, USA) according to the 4500-P standard at 420 nm [40]. Concentrations of Cl were determined by a potentiometric precipitation titration according to NP-831/1970. Briefly, a silver nitrate standard solution (0.1 mol/L) was standardized with a solution of sodium chloride (0.1 mol/L). Titrations were performed with a Metrohm 780 pH-meter (Herisau, Switzerland)), a silver titrode (Metrohm), a Metrohm 715 Dosimat buret and a magnetic stirrer, as previously described by Plácido et al. [44]. The end point of each titration was determined by using the second derivative of the titration curve. All measurements were done in triplicate.

Standard calibration method was used to prepare daily calibration curves (*n* ≥ 6) for each element. All calibration curves showed good linearity over the entire range of concentrations with acceptable quadratic correlation coefficients (R^2^ ≥ 0.999). Detection and quantification limits were calculated as the minimum detectable amounts of analyte with a signal-to-noise-ratio of 3:1 [45]. Detection limits varied between 3.5 µg/kg for Cu to 5.8 mg/kg for P, with corresponding quantification limits ranging between 12 µg/kg to 20 mg/kg. Inter- and intra-assay precision were evaluated through relative standard deviation with values ranging between 0.40–2.1% and 1.3–10%, respectively. Global precision values followed the recommendation of International Union Of Pure and Applied Chemistry (IUPAC) [46]. Regarding accuracy, the analytical methodology was validated by systematic recovery experiments performed at different fortification levels and using at least two different samples from each type of bread. Bread samples were previously fortified before drying and digestion procedures and the achieved recovery values ranged between 73% (Mn) to 107% (Fe). Blank MAD extracts and mineral standard solutions were daily and regularly analyzed between samples to check instrument performance. Mineral analyses were done in triplicate. Concentrations were determined on both wet and dry weight bases however to simplify the discussion of results levels are only presented on ww basis.

### 3.4. Statistical Analysis

Statistical analysis was performed using the SPSS (IBM SPSS Statistics 20, IBM, Armonk, NY, USA) and Statistica software (v. 7, StatSoft Inc., Tulsa, OK, USA). Data were expressed as median, 25 and 75 percentiles and range (minimum–maximum). Comparison between the mineral content among the different bread samples were performed by non-parametric tests (Krushkal–Wallis and Mann–Whitney U-test) since normal distribution of the data was not observed. Statistical significance was defined as *p* ≤ 0.05 (*p*-value at 95% confidence level). Principal Component Analysis (PCA) was performed as pattern recognition technique for the four types of bread.

## 4. Conclusions

The chemical composition of four types of highly consumed Portuguese breads was characterized concerning the levels of six macro (Ca, Mg, Na, K, P, and Cl) and four microelements (Cu, Fe, Mn, and Zn). The regional Broa de Avintes bread, prepared with maize and rye flours, presented the highest content of all macro (except P and Cl) and microminerals compared with the other three types of bread under study. Daily consumption of bread contributes to an equilibrated diet since bread is rich in several essential minerals ranging from 0.21 mg/100 g of Cu (wheat bread) to 537 mg/100 g of Na (maize/rye bread). Maize/rye bread can be a valuable source (more than 15% of RDA value) of Mg, Na, K, P, Cu, Fe, Mn, and Zn while the white wheat bread presented the lowest intakes of Cu and Zn. However, special attention needs to be taken with the daily ingestion of Na since the consumption of 100 g of bread represented an intake of 422–537 mg; these values are only slightly lower than 550 mg, which is the maximum concentration allowed by the Portuguese legislation [30]. The consumption of bread represented a median intake of more than 28% of RDA of Na (28% in maize bread to 36% in maize/rye bread). Therefore, national actions should be implemented to monitor and control the amount of sodium chloride added to bread and preventive programs should be introduced to help food industry in the reduction of salt from all foods without losing their sensorial value.

The PCA model was constructed with the concentrations of eight elements (Mg, Ca, K, P, Cu, Fe, Mn, and Zn) and allowed the differentiation among three types of bread (white wheat, maize, and maize/rye bread). Zn, Mg, Mn, Fe, P, K, Cu, and Ca proved to be good chemical markers to discriminate different types of bread. Further investigation is required with other bread compositions to explore deeply the achieved findings.

## Figures and Tables

**Table 1:** Review of bread mineral content (mean ±SD and range; mg/100 g wet weight (ww)) reported in the literature (2000–2017).

Bread Type *	Continent, Country	Notes	Ca	Mg	Na	K	P	Cu	Fe	Mn	Zn	Study
			Mean ± SD (Range) mg/100 g Sample									
Wheat	Africa, Egypt	Prepared	29.1	29.8	NR	86.1	87.3	0.23	1.75	NR	0.64	[17] *
	Africa, Ethiopia	Market (*n* = 5)	23.1 ± 3.1 (20.2–26.3)	NR	NR	NR	182 ± 9 (174–189)	NR	5.4 ± 1.2 (3.8–7.4)	NR	1.60 ± 0.24 (1.17–1.84)	[25]
	Africa, Nigeria	Prepared	249 ± 0.40	156 ± 0.02	16 ± 0.02	NR	NR	NR	33.5 ± 0.02	NR	6.7	[8] *
	America, Brazil	(*n* = 15)	2.5	NR	NR	NR	105	NR	5.1	NR	1.7	[18]
	America, USA	Market	104	46	528	200	152	NR	3.28	NR	1.2	[27] *
	America, Chile	Market (*n* = 21)	NR	NR	NR	NR	NR	0.08 ± 0.03	1.39 ± 0.20	NR	0.57 ± 0.15	[22]
	Asia, India	(*n* = 3)	11.6	27.9	NR	NR	NR	NR	1.20	NR	0.20	[24] ^#^
	Europe, Bulgaria	Prepared	23	27	NR	NR	NR	NR	1	NR	1	[1] *
	Europe, Finland	Market (*n* = 1)	41.3	26.1	NR	137.6	103.2	0.14	1.1	0.6	0.8	[20]
	Europe, Poland	Prepared (*n* = 10)	31; 34	20; 29	345; 344	176; 232	116; 169	NR	1.48; 2.08	NR	1.10; 1.59	[28]
	Europe, Poland	Corn, wheat starch	19.2 ± 2.8	15.0 ± 1.0	770 ± 21	74 ± 12	NR	0.01	0.38 ± 0.04	<0.01	0.29 ± 0.03	[9] *
	Europe, Portugal	Market (*n* = 18)	NR	NR	465 ± 109 (280–581)	NR	NR	NR	NR	NR	NR	[26]
	Europe, Portugal	Market (*n* = 5)	43	31	610	121	162	NR	2.2	NR	1.0	[21]
	Europe, Portugal	Market (*n* = 3)	NR	NR	684 ± 4.0	155.9 ± 1.3	NR	NR	NR	NR	NR	[6]
	Europe, Serbia	Prepared	45.9	6.6	NR	170 ± 13	65.6 ± 6.6	0.09 ± 0.02	1.14 ± 0.12	NR	0.27 ± 0.11	[23] *
	Europe, Spain	Market, prepared	24.7	2.5	24.2	6.3	NR	0.01	0.07	0.03	0.05	[13] *
	Europe, Spain	(*n* = 3)	23.3	NR	NR	NR	NR	NR	1.13	NR	1.53	[15] ^#^
	Europe, Spain, Poland	Prepared	19.0	17.8	NR	115	77.7	0.14	1.15	0.39	0.71	[16] ^#^*
	Europe, Turkey	(*n* = 2)	22.5	32.4	NR	205.2	236.8	0.27	1.69	0.31	1.4	[14]
	Europe, Turkey	Prepared (*n* = 3)	30.2	NR	NR	266	135	NR	1.88	NR	0.47	[19]
Maize	Africa, Ethiopia	Market (*n* = 16)	14	NR	NR	NR	NR	NR	3.4	NR	1.66	[12]
	Africa, Ethiopia	Market (*n* = 4)	8.3 ± 1.4 (6.4–9.5)	NR	NR	NR	176 ± 8 (170–181)	NR	5.2 ± 1.2 (4.4–6.8)	NR	1.10 ± 0.30 (0.83–1.10)	[25]
	Europe, Portugal	Market (*n* = 5)	14	37	282	178	109	NR	1.3	NR	0.4	[21]
	Europe, Portugal	Maize/wheat Market (*n* = 3)	NR	NR	629 ± 9.0	253 ± 0.7	NR	NR	NR	NR	NR	[6]
Rye	America, USA	Market	312	187	219	NR	NR	NR	5.6	NR	6.9	[27] *
	Europe, Finland	Market (*n* = 1)	19.8	73.9	NR	306.5	168.3	0.3	2.0	1.7	1.7	[20]
	Europe, Poland	Market (*n* = 6)	37.1–106.5	86–106	NR	NR	293–456	0.31–0.42	2.74–3.95	2.17–3.68	2.11–3.98	[10]
	Europe, Portugal	Market (*n* = 3)	NR	NR	486 ± 61	172.6 ± 3.3	NR	NR	NR	NR	NR	[6]
	Europe, Portugal	Market (*n* = 5)	56	62	517	248	103	NR	1.2	NR	1.3	[21]
	Europe, Portugal	Market (*n* = 9)	NR	NR	521 ± 71 (453–586)	NR	NR	NR	NR	NR	NR	[26]
Sorghum	Africa, Nigeria	Prepared	6.6–13.2	68.7–121	NR	126–233	NR	NR	NR	NR	NR	[29] *

NR: not reported; ^#^ Data were converted to wet weight basis to allow direct comparison. * When the number of samples (*n*) is not indicated, it means that it was not reported.

**Table 2:** Recommended daily allowance (RDA; mg/day)/adequate intake (AI; mg/day) and estimated daily mineral intake (DMI, %) of each element for the consumption of 100 g of bread.

Element	RDA/AI ^a^ (mg/day)	DMI (%)			
		Wheat Bread	Maize Bread	Maize/Wheat Bread	Maize/Rye Bread
Mg	375	11 (6.2–20)	22 (14–40)	8.1 (3.9–18)	32 (27–37)
Ca	800	1.8 (1.2–5.8)	1.6 (0.7–2.7)	1.7 (1.1–2.5)	3.0 (2.6–3.7)
Na	1500	32 (23–54)	28 (14–47)	30 (25–33)	36 (24–52)
K	2000	10 (4.9–18)	8.3 (5.4–19)	11 (4.4–13)	20 (18–22)
P	700	27 (12–38)	16 (11–25)	18 (14–32)	16 (13- 21)
Cu	1	21 (9.1–32)	22 (11–32)	24 (11–38)	32 (26–37)
Fe	14	12 (6.8–19)	18 (7.3–27)	11 (9.1–23)	22 (18–30)
Mn	2	60 (27–103)	69 (28–118)	57 (38–118)	181 (127–201)
Zn	10	11 (7.0–18)	18 (11–30)	12 (8.0–22)	24 (20–27)
Cl	800	4.8 (3.1–6.4)	4.4 (3.0–5.8)	4.8 (2.8–5.4)	3.3 (2.4–4.1)

^a^ According to [33,34].

**Table 3:** Optimized experimental conditions used in High-Resolution Continuum Source Atomic Absorption Spectrometer (HR-CS-AAS) with atomization by flame (Ca, Mg, Na, and K) and graphite furnace (Cu, Fe, Mn, and Zn).

**HR-CS-AAS-FlameA**	**Ca**	**Mg**	**Na**	**K**
Wavelength (nm)	422.6728	285.2125	588.9953	766.4905
Height of burner (mm)	5	8	6	8
Acetylene flow (L/h)	50	65	90	60
Acetylene/air flow (L/h)	0.19	0.14	0.20	0.24
**HR-CS-AAS-Graphite Furnace Program**	**Cu, Fe**	**Mn, Zn**		
	T(°C); ramp time (s); hold time (s)			
1st drying	80; 6.0; 20	80; 6.0; 20		
2nd drying	90; 3.0; 20	90; 3.0; 20		
3rd drying	110; 5.0; 10	110; 5.0: 10		
Ashing	1300; 300; 10	1200; 300; 10		
Atomization	2000; 1500; 4.0	2000; 1500; 3.0		
Furnace cleaning	2450; 500; 4.0	2450; 500; 4.0		
